# Systematic review of challenges and prospective recommendations of medically assisted reproductive technology in developing countries

**DOI:** 10.3389/frph.2025.1678033

**Published:** 2025-11-27

**Authors:** Melese Dereje Mesfin, Dahabo Adi Galgallo, Leman Atmaca, Kálmán András Kovács, Ákos Várnagy, Viktória Prémusz

**Affiliations:** 1 Doctoral School of Health Sciences, Faculty of Health Sciences, University of Pécs, Pécs, Hungary; 2 College of Medicine and Health Sciences, Wolkite University, Wolkite, Ethiopia; 3 Department of Health, Marsabit, Kenya; 4 National Laboratory on Human Reproduction, University of Pécs, Pécs, Hungary; 5 Department of Obstetrics and Gynaecology, Medical School, University of Pécs, Pécs, Hungary; 6 Directorate for Human Reproduction, National Directorate General for Hospitals, Budapest, Hungary; 7 Faculty of Health Sciences, University of Pécs, Pécs, Hungary

**Keywords:** assisted reproductive technologies (ART), challenges, recommendation, barriers, *in vitro* fertilization, developing countries, intracytoplasmic sperm injection

## Abstract

**Introduction:**

Infertility is a global public health issue which affects significant portion of adult population. In developing nations, infertility has long been disregarded as a reproductive health problem. Despite being available for almost for five decades, most people in resource-poor nations still face challenges to access assisted reproductive technology. We conducted this systematic review to explore the reasons behind such gaps and solutions outlined to address them.

**Methods:**

After a study protocol was registered with the International Prospective Register of Systematic Reviews (PROSPERO), we conducted a comprehensive search using predefined keywords and medical subject headings across major electronic databases: - Ovid MEDLINE, PubMed, Web of Science, and Google Scholar.

**Results and Discussion:**

The database search resulted in total of 3097 citations; after removal of 2256 duplicates, 765 citations were selected for title and abstract review. A full text review was conducted on the 137 articles 43 studies were included in the final analysis. The majorly cited challenge was the high costs associated with treatment, followed by accessibility and infrastructural obstacles, psychosocial and cultural barriers including the unacceptance of a baby born from assisted reproduction. Absence of supportive policies coupled with religious factors worsen the problem. Governmental, non-governmental, and international organizations should collaborate to address affordability and accessibility issues and to resolve socio-cultural and religious challenges through the inclusion of infertility treatment in the existing health system, revisiting the financial mechanisms, and creating supportive policies in partnership with community and religious leaders.

**Systematic Review Registration:**

https://www.crd.york.ac.uk/PROSPERO/view/CRD42025632094, PROSPERO CRD42025632094.

## 1 Introduction

Infertility is a disease of the male or female reproductive system that is characterized by inability to conceive a child after at least 12 months of unprotected sexual intercourse (1). Globally, male factors account for about 40% of infertility in couples, while female factors account for another 40%. The remaining 30% is attributed to both or unidentified reasons (2, 3). According to a 2023 report by the World Health Organization (WHO), infertility is a growing public health issue, with involuntary childlessness affecting about 17.5% of the adult population globally (4).

The most common causes of infertility include infections from unsafe abortion, complications during childbirth, and tubal disease from sexually transmitted infections. The prevalence of infertility is higher in developing countries, particularly in Africa and most of the involuntary childlessness in Africa is related to tubal factors which reaches up to 85% compared to 33% globally (2, 3, 5, 6).

Infertility problems are not limited to gynecological and andrological aspects; they also represent a serious health concern since they often impair couples and individuals psychological, financial and social well-being (7). Infertility is viewed as a serious life issue with long-term effects and it has a significant negative psycho-social impact on couples who live in a developing nation where almost every society is a highly pronatalist (8–12).

In poor economic settings like sub-Saharan Africa (SSA), the importance of prevention is the most effective strategy due to the presence of many avoidable causes of infertility and limited resources for treatment; however, the need for treatment remains undeniable despite those constraints. Infertility treatment should precisely target the recognized cause of infertility by employing methods that improve the chances of getting pregnant and delivering a healthy baby (11, 13, 14).

Assisted reproductive technology (ART) can be defined as any treatment or procedure that utilizes sperm, human oocytes or embryos to generate pregnancy *in vitro* (15). ART has been around for the last forty years. Over this time, it has given many people who previously struggled with delivery hope but unfortunately, ART affordability and accessibility challenges still continue to be issues in many contexts. Since 2001 WHO has promoted the consideration of infertility as a worldwide public health issue and the wider application of ART to address infertility in developing countries (16–18).

There were only 15 (31%) IVF centers in SSA countries until 2010, which is a small number when compared to those in Asia, the Middle East, and Latin America, except for the following four SSA nations: Egypt with thirty-five IVF centers, Nigeria with up to twenty, South Africa with up to fifteen, and Ghana with seven IVF centers which can be considered as comparative regional success stories (11, 19, 20).

The 2010 International Federation of Fertility Societies (IFFS) monitoring report completely omitted a number of geographical locations. The report, for example, made no mention of any of the major Central Asian nations, including Kyrgyzstan, Uzbekistan, Tajikistan, Afghanistan, Kazakhstan, Mongolia and Turkmenistan which is particularly concerning considering the fact that majority of Central Asia has the highest rate of secondary infertility globally (21–24).

Even though decades have passed since the introduction of medically assisted reproductive technologies in to developing countries, there are still several challenges associated with the delivery of infertility-related care. We conducted this systematic review to illustrate and summarize t major challenges associated with ART in terms of availability, accessibility, affordability acceptability, cultural, normative and ethical plausibility, Political and policy priority in developing countries and to outline prospective recommendations provided by various studies to address those challenges.

### 1.1 The following questions has been answered by the systematic review

What is the current state of assisted reproduction in developing nations?
What are the major challenges associated with assisted reproductive technologies?
What are the major recommendations outlined to address challenges associated with assisted reproductive technologies?

## 2 Methods and materials

We registered the review protocol with the International Prospective Register of Systematic Reviews (PROSPERO) (registration number CRD42025632094) and published it on January 22, 2025. Throughout the systematic review, we followed Preferred Reporting Items for Systematic Reviews (PRISMA) guidelines. No ethical approval was requested since the study doesn't involve any human contact.

### 2.1 Selection criteria

The search was conducted after the selection criteria were specified. Articles meeting the pre-specified Population, intervention, and outcomes (PICO) criteria, such as studies which include infertility clients, health professionals, health policymakers, and fertility-related service providers etc. as their populations (P); Medically assisted reproductive technologies such as IVF, ICSI, ovarian stimulation (OS), Gamete intrafallopian transfer (GIFT)… as the intervention (I); and studies that outline in their outcomes (O) as barriers to medically ART in terms of either availability, affordability, accessibility, utilization, socio-cultural, legal, and ethical challenges in developing countries both quantitatively and qualitatively were selected for this systematic review.

### 2.2 Search strategy

Using predefined keywords and medical subject headings, we performed a comprehensive search across major electronic databases of PubMed, Ovid MEDLINE, EMBASE, Google Scholar, and Web of Science.

The pre-defined search strategy included keywords and medical subject headings (MeSH) terms. Key words for outcome of the study or challenges (O) include:- barrier* OR hindrance* OR obstacle* OR hurdle* OR impediment* OR limitation* OR availability* OR accessibility OR usability OR approachability OR presence OR openness OR utilization OR use* OR employment OR application* OR exploitation OR operation OR consumption OR deployment OR implementation OR exercise OR engagement OR reachability OR attainability etc …. Key words and MeSH terms for intervention ART (I) include:- “assisted reproductive technology”, “assisted reproductive technic*” OR “Assisted Reproductive Technologies” OR “Embryo transfer” OR Maturation OR “Fertility preservation” OR “Gamete Intrafallopian Transfer” OR “Sperm Retrieval” OR “Oocyte Retrieval” OR “Zygote Intrafallopian Transfer” OR “Oocyte Donation” OR “*in vitro* Fertilization” OR “*in vitro* Fertilization*” OR “Intra cytoplasmic sperm injection*” OR IVF* OR ICSI* etc… Key words for Population (P) or developing countries included “Developing Countries” OR “developing country” OR “developing countries” OR “developing nation” OR “developing nations” OR “developing population” OR “developing populations” OR “developing world” OR “less developed country” OR “less developed countries” OR “less developed nation” OR “less developed nations” OR “less developed population” OR “less developed populations” OR “less developed world” OR “lesser developed country” OR “lesser developed countries” OR “lesser developed nation” OR “lesser developed nations” OR “lesser developed population"[tiab] OR “lesser developed populations” OR “lesser developed world” OR “under developed country”[tiab] OR “under developed countries” and named list of developing countries according to world population review report 2025 (25) combined with Boolean operator” OR”. Furthermore, additional keywords from search strategies of previously published related reviews were used to further strengthen the search output. For detailed search strategy please refer Supplementary File S1, Table 1.

**Table 1:** Summary of articles included in the systematic review of ART challenges in developing countries.

S.no.	References	Country/Region	Population	Study design	Major challenges associated with assisted reproductive technology						
					Affordability/Cost	Accessibility/Infrastructural	Socio-cultural	Ethico-legal	Religious	Political	Awareness
1	Kyei et al. (31)	Ghana	Infertility Clients	Qualitative	√	√	√				
2	Dyer et al. (54)	Africa	Art Centers	Data registry		√				√	
3	Souza (29)	Brazil	Art Centers	Qualitative	√						
4	Garcia & Bellamy (32)	Brazil	Art Centers	Qualitative	√	√		√	√		
5	Hiadzi et al. (33)	Ghana	Infertility Clients, Providers	Qualitative	√	√					
6	Appiah & Ganle (34)	Ghana	Art Professionals	Qualitative	√	√	√	√			
7	Bezad et al. (44)	Morocco	Policymakers, Health Actors	Qualitative						√	
8	Makuch et al. (55)	Brazil	Practitioners, infertility clients	Qualitative	√	√					
9	Afferri et al. (35)	Gambia	Policymakers, Practitioners	Qualitative Survey	√	√				√	
10	Bennett et al. (39)	Indonesia	Infertility Clients	Quantitative survey		√					
11	Botha et al. (27)	SSA	Clients, Registries	Systematic review	√					√	
12	Chiware et al. (38)	LMIC	Clients, Registers	Systematic review	√	√					
13	Njagi et al. (30)	LMIC	Provides, Clients	Systematic review	√					√	
14	Fizazi et al. (37)	Algeria	Infertility Clients	Quantitative survey	√						
15	Purvis (51)	Indonesia	Provides, Clients	Review	√						
16	Asante-Afari et al. (71)	Ghana	Infertility Clints	Qualitative Survey	√				√		
17	Inhorn (56)	Egypt	Provides, Clients	Review			√				
18	Okafor et al. (57)	Nigeria	Infertility Clients	Cross-sectional	√					√	
19	Akande et al. (72)	Nigeria	Infertility Clients	Cross-sectional	√						√
20	Tholeti et al. (58)	India	Provides, Clients	Review	√						
21	Barnes et al. (47)	Ghana	Professionals, Managers,	Qualitative		√		√		√	
22	Anaman-Torgbor et al. (73)	Ghana	Infertility Clients	Qualitative	√		√				
23	Murage et al. (16)	Kenya	Obstetricians, Gynecologists	Cross-sectional	√	√					
24	Shahin (74)	Egypt	Infertility Clients	Quantitative survey	√						
25	Makuch & Bahamondes (40)	Brazil	Health Authorities	Mixed Methos	√	√				√	
26	Ombelet et al. (70)	Developing countries	Professionals, Clients	Systematic review		√				√	
27	Ma et al. (59)	China	Registries	Cross-sectional	√	√					
28	Okantey (75)	Ghana	Infertility Clients, Professionals	Qualitative	√		√				
29	Ezeome et al. (46)	Nigeria	ART Clients	Qualitative	√		√				√
30	Whittaker et al. (60)	South Africa, Zimbabwe,	ART Specialists, Gynecologists	Qualitative	√	√				√	
31	Z et al. (76)	Malaysia	Infertility Clients, Professionals	Qualitative					√		
32	Bittaye et al. (41)	Gambia	Professionals, Students	Quantitative survey		√		√	√	√	
33	Majangara Karaga et al. (77)	SSA	Clinicians, Authorities	Mixed Methos	√					√	
34	Oti-Boadi et al.) (62)	Ghana	Infertility Clients	Qualitative Survey	√		√				√
35	Dewi et al. (69)	developing countries	Provides, Clients	Scoping review		√	√		√		√
36	Ranjbar et al. (64)	Iran	Infertility Clients	Qualitative			√				
37	Chikeme et al. (78)	Nigeria	Infertility Clients	Cross-sectional Survey	√	√				√	√
38	Widge and Cleland (42)	India	Gynecologists	Cross-sectional survey		√				√	
39	Afferri et al. (79)	Gambia	Health Facilities	Cross-sectional study	√	√				√	
40	Binarwan Halim E.G. et al (80)	Indonesia	ART Clients	Quantitative survey	√	√					
41	Gerrits & Shaw (81)	SSA	Professionals, Clients	Systematic review		√	√				
42	Dyer et al. (36)	South Africa	ART Clients	Observational follow-up study	√					√	
43	Njogu et al. (66)	Kenya	ART Clients	Qualitative	√		√				

ART, Assisted reproductive technology; LMIC, low- and middle-income country; ART, medically assisted reproductive technology; SSA, Sub-Saharan Africa.

### 2.3 Inclusion and exclusion criteria

We included peer-reviewed quantitative, qualitative, mixed method studies and reviews which were conducted in English language. Studies conducted in developing countries, focused on barriers and challenges associated with assisted reproductive technology. No restriction was applied on year of publication and for any published articles we contacted authors for full texts. To access more articles we reviewed the reference lists of relevant publications.

We excluded p conference papers, opinion-based papers, commentaries, papers without original data, and other non-peer-reviewed publications. In addition, studies conducted in developed countries were not included in the review. Throughout the review process, the authors used Rayaan and endnote software for the removal of duplicates, exclusion, inclusion, and organization of articles (26, 27).

### 2.4 Data extraction

Three reviewers (DM, LA, and DG) independently examined the studies, discrepancies were discussed, compared, and resolved by the reviewers based on the explicitly set inclusion and exclusion criteria. When disagreements can't be resolved by through discussion among reviewers we involved the fourth reviewer (VP) for resolving disagreements among the reviewers and to make the final decision. We organized the extracted data from relevant studies in a data extraction sheet for final synthesis. Information about the primary author, publication time, nation or region of the study, population, study design, sample size, research question, and outcomes were among the extracted data from the studies. In the final analysis we included only studies with full texts.

### 2.5 Risk of bias assessment

In order to reduce selection bias, we blinded all the reviewers as much as possible during the review of each paper; we relied on the independent evaluation of the three reviewers. In addition, the reviewers used a critical appraisal checklist, such as the Critical Appraisal Skills Programme (CASP) checklists (28), to determine the quality, relevance, and eligibility of the studies. CASP is a tool that enables one to methodically evaluate the reliability, applicability, and outcomes of published studies. And finally, all discrepancies were resolved by consensus, and only those studies with none of the signaling issues were considered to have a low risk of bias and included in the review.

### 2.6 Data analysis

This study investigated the common challenges concerning ART in developing countries. Findings from a systematic review were highly heterogeneous and comprised a wide range of settings, methods, and results, and hence, the authors used narrative analysis for data synthesis. Eligible studies were grouped according to outcome themes, such as Financial challenge, Accessibility and infrastructural challenges, Policy and political challenges, Socio-cultural challenges, Ethico legal challenges and religious challenges faced by clients of assisted reproductive technology.

## 3 Results

**Figure 1:**
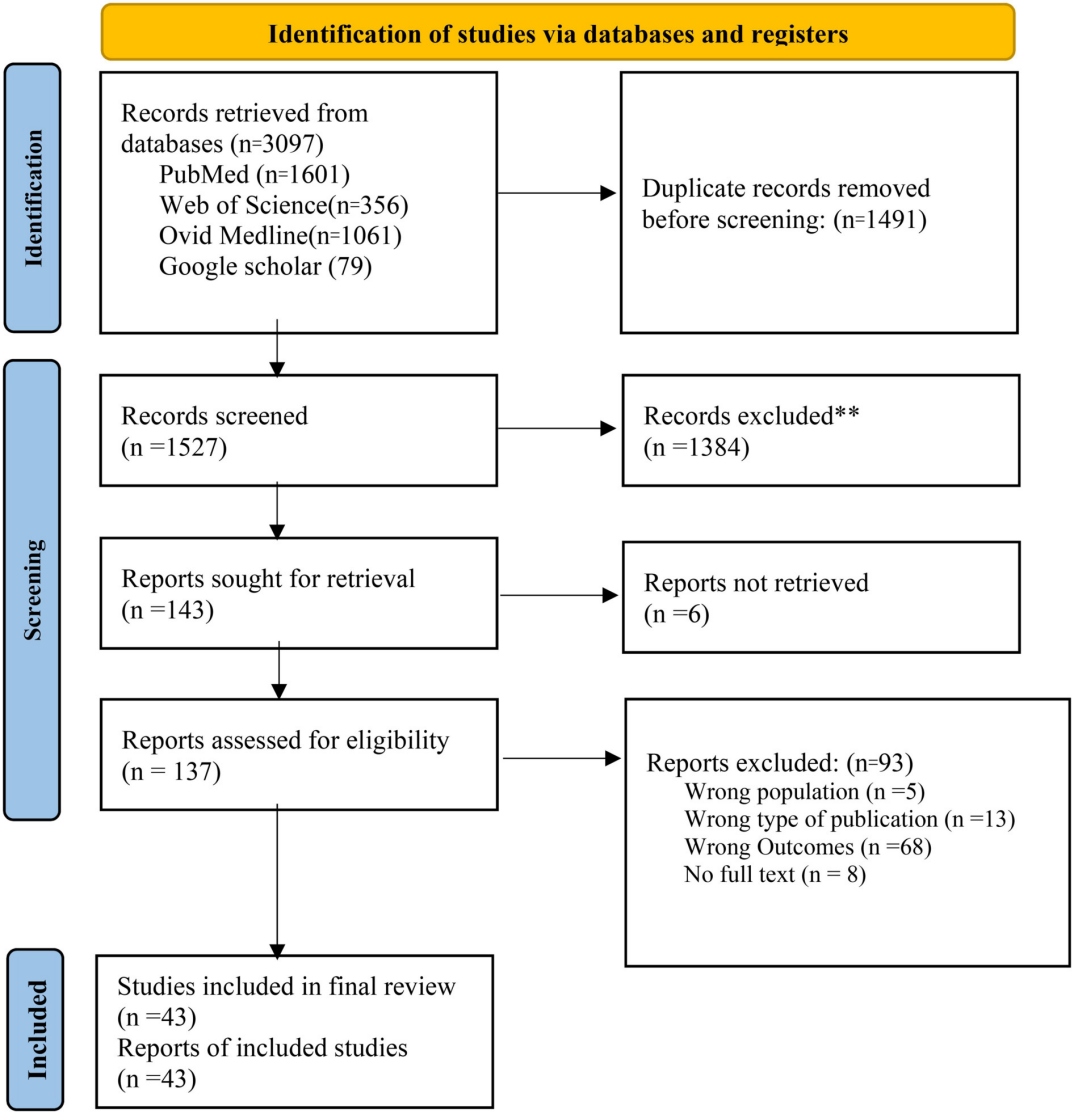
PRISMA Flow diagram of included and excluded articles in systematic review of challenges and prospective recommendation of Medically Assisted Reproductive Technology in developing countries.

An Extensive search of electronic databases resulted in 3,097 citations. After the removal of 2,256 duplicates, the authors selected 765 citations for title and abstract review, which resulted in 137 citations. Second to the title and abstract review, we conducted a full-text review on 137 articles and excluded 96 citations for various reasons. duplicate entries from the database searches, studies conducted in developed countries, non-relevant studies, wrong outcomes, inability to access the full-text document, and not being the right type of publication were among the reasons for exclusion of articles. Additionally, we conducted a snowball search on google scholar to look for additional articles based on the reference lists of previously extracted articles, which resulted in 79 studies, from which 73 articles were reviewed and removed due to duplication and eligibility, and 3 of them were included in the final review. Finally, through the above process, we extracted 43 articles for analysis and the whole review process was conducted following Preferred Reporting Items for Systematic Reviews (PRISMA) guidelines (see Figure 1).

About the characteristics of the retrieved studies, nineteen of them were quantitative observational surveys, twenty-two of them were qualitative studies, seven of them were reviews, one was a registry, and the rest were mixed-methods studies. Majority of the studies were conducted mainly in the in Africa (25), Asia (6), and Latin America (4), and the most commonly mentioned challenges were Cost and affordability as mentioned by (32), accessibility and infrastructure by (26) studies, Policy and Political challenges by (16) articles and the rest were Socio-cultural, religious, Awareness and Ethico-legal challenges as stipulated by (15), (5), (5) and (4) articles respectively. Financial or high cost of treatement and infrastructral challenges are common challenges globally whereas Soci-cultural, Religious and Political challenges are majorly indictaed in Africa and Asia for more detailled presentataion please refer Supplementary Figure S2, Table S3.

### 3.1 Financial challenges

Unaffordability of ART service by the general population and the associated postponement of treatment and catastrophic expenditure, which led to poor financial recovery, were the commonly mentioned challenges by most articles. The absence of any form of financial protection for clients, either as part of mandatory national insurance or private insurance, was another obstacle in the area. The direct medical expenses that infertility clients pay for treatment are also significantly greater than the GDP per capita and the annual average income of clients, furthermore in most developing countries ART service is dominated by a private sector with a little control and regulation of the government which sometimes leads to moral hazard with raged to the cost of service among the providers (29–37).

### 3.2 Accessibility and infrastructural challenges

The other most common challenge associated with utilization of ART service is the inaccessibility of ART centers; most of the infertility centers are concentrated in capital cities, and people outside the capital have to travel long distances and look for an accommodation. Furthermore, the long waiting list to access the service made it difficult for clients who want to use the service. In addition to that lack of appropriate training among health professionals and absence specialized training course in the medical curricula regarding ART are among the factors affecting the quality of the service (38–42).

### 3.3 Policy-political challenges

The issue of infertility is not a main political agenda in developing countries due to the high level of focus directed towards prevention and control of infectious causes of disease, population control, maternal and child health services which led to the absence of a national-level program, plan and priority for infertility-related services. In order to guarantee universal access without discrimination and to protect and promote the human rights of all parties concerned, WHO proposes the creation of enabling laws and regulations that govern third-party reproduction and ART (27, 43–45).

### 3.4 Socio-cultural and psychological challenges

Negative cultural, religious, and social constructs associated with infertility are another major challenge associated with acceptance and utilization of ART in developing nations specially in African counters. the high value linked with having a child among the highly pronatalist society of developing countries and associated stigmatization against childless couples exposed infertile couples to an unimaginable level of psychological distress, which can never be justified by the high level of population in those nations (31, 34, 46).

### 3.5 Ethico-legal challenges

In undeveloped nations, there is often little oversight of fertility practices which could expose infertility clients to expensive and poor medical treatment which might lead to unintended consequences. Furthermore, the absence of clear rules, regulations, and policies regarding infertility care and the minimum attention directed towards it created a condition where privatization, low-quality service, commercialization, unjustifiably high costs and unnecessary repetition of investigations are prominent among providers of assisted fertility service providers (16, 32, 34, 47–49).

### 3.6 Religious challenges

Religious views are among the factors that prevented certain developing countries from fully implementing assisted reproduction and they were mostly prompted by concerns about the moral status embryo. In some countries Islamic laws govern clinical practice and effectively prohibit the use of some techniques. For example, homosexual couples are unable to obtain ART since the Qur'an denounces of homosexual relationships (50–53).

The above listed problems call for timely, integrated, and multifaceted action in addressing multitudes of challenges associated with the delivery of reproductive services in developing countries. For further presentation of recommendation see Supplementary Table S2.

## 4 Discussion

Despite the widespread introduction of medically assisted reproduction globally, there are still multiple sets of challenges in the developing world to make the service available, accessible, and usable for clients in need of the service.

As indicated by the majority of the research, the major challenge was the high cost of ART large proportion of the low-income society could not afford the expense of infertility treatment. Furthermore, ART in some developing countries is typically dominated by the private sector as government support remains limited, which by itself leads to a high cost of treatment cycles and proliferation of ART clinics that somehow commercialized the services as observed by prescribing unnecessarily repeated tests, low-quality service and a low treatment success rate (27, 29–32, 34, 35, 37–39, 51, 54–57).

Large proportion of childless couples in developing countries are unable to get infertility treatment services due to a lack of insurance coverage, financial protection and an absence of allocated budget for such procedures (35, 58). If we take Algeria as an example the National Social Security Fund's failure to reimburse infertility therapy made the service to be outrageously costly, which makes it highly unaffordable for infertile couples with moderate to low incomes (37).

Another challenge to accessing assisted reproductive treatment emanates from infrastructural issues such as physical distance of the treatment centers from the patient's living address: Infertility clients have to travel from their homes or the surrounding areas to the fertility centers, which are mostly located in capital cities. As a result, they occasionally had to find lodging in a city where the fertility center is located, which is costly and out of reach for the majority of clients. A study from Brazil indicated that 83% of health authorities who participated in a study reported the absence of ART within the unified health system (SUS). Furthermore, lack of political commitment and shortage of appropriate resources and trained professionals to implement ART services are forcing people who need the service to travel long distances and go through long waiting times. Distance was a major obstacle to accessing assisted reproduction centers in South Africa, long commutes are required for infertility clients who live in Rural cities to access ART service and there were 354.9 (25.4%) million people living in 148 Chinese cities without access to an ART facility as of 2018 (31, 40, 59, 60). A study from Indonesia furthe indicated that more than 54% of infertility clients had to commute long distances to receive care since infertility services are not evenly distributed geographically and concentrated in large cities, like Java According to the survey conducted in east African nation Kenya, despite the high level of tubal disease prevalence, only three assisted reproduction centers were available in the country and after an investigation by local health facilities, clients are commonly referred to infertility centers found in other countries sometimes as far away as South Africa or India. This maldistribution and geographical inaccessibility of service have more negative consequence apart from cost, they also imply the treatment outcome of the clients. a study by Rodrigues-Martins et al. indicated that geographic disparities have significant influence on the treatment outcomes and affect implantation success, miscarriage rate and fertility rates (16, 39, 61).

Support and funding for assisted reproduction services are required from governmental, non-governmental, and international organizations to address the challenges associated with accessibility. Usage of modest stimulation regimens and oocyte retrievals to lower drug expenses was also another recommendation. Cost sharing by private and state-owned health insurance firms for assisted reproductive services is also mentioned as a prospective solution to lower the cost of fertility treatment (29, 31).

In the majority of developing countries, significant proportion of medical personnel lack proper training in infertility, there is no recognized degree program, ART specialty, or regular medical training (41, 42). For instance, about 46% of Indian medical experts who responded to a survey agreed that a lack of specialized training was one of the difficulties in treating infertility. According to the new National Health Policy document of Gambia lack of appropriate training was mentioned as one of the obstacles to implementing fertility treatment (35, 42). Health professionals’ skills must be updated through specialized fertility care training in order to deliver standardized ART services. To further increase the quality of services, advanced training centers for ART must be established, and the topic shall even be added to medical training curricula Establishing accreditation organizations and defining precise training standards and qualifications for practitioners are necessary to preserve the standard of current and future assisted reproductive treatments (35, 42, 60).

Establishing guidelines, policies and rules to protect the health of couples receiving treatment is crucial to the delivery of fertility care. The absence of guidelines and regulations governing medically assisted reproduction is even more problematic since it has been shown that even countries with guidelines have gaps in effectively handling ethical, social, and legal aspects of this crucial field of medicine. This challenges were practically observed in nations, like Brazil and India, who lack regulations for governing assisted reproductive services (32, 33, 42). It is recommended that the government and other relevant parties should work towards passing the necessary legislation, creating a regulatory agency and defining ethical standards for ART practice in order to address the ethical and legal issues that arise in the practice (33, 34, 47–49).

Cultural perceptions about infertility services posed a palpable challenge to the use of ART. Participants in in different studies claim that ART procedures contradict with their cultural beliefs, which typically and solely link male-to-female sexual interaction with child birth. These beliefs may discourage people from seeking ART when natural conception fails. The difficulty of finding surrogate mothers is also another major cultural obstacle cited by participants (62, 63).

Studies from Iran and Ghana showed that women undergoing ART treatment suffered from anxiety, stress and frustration during treatment. The aforementioned socio-cultural and psychological challenges highlight the need for community-level ART education and sensitization since health Literacy have a significant influence on women reproduction and furthermore psychosocial therapy needs to be incorporated into ART treatments to lessen the psychological distress among the clients (64–67).

Religion was another common challenge cited by reviewed articles towards access of assisted reproduction and this challenges are not limited to specific religious group but rather they are existent in majority of global religious groups from Muslim to Catholicism to Further put this with an example bin the Sunni Muslim world, which includes the majority of Arab nations, the use of third party assisted reproduction technique is forbidden by religious decrees, legislative provisions and professional and bioethical standards of medical practice. Similarly, Roman Catholicism considers ART to be wholly undesirable, but Anglicans, Protestants, and Coptic Christians embrace the majority of ART forms that don't need gamete or embryo donation (50–53). In order to promote diversity and offer individualized pre-ART counseling, it is crucial to recognize how religion influences ART beliefs and use. The aforementioned conditions highlight the significance of tailored health education to address those particular religious issues and the need to work with religious leaders and community representataives opinion leaders in order to address those challenges and to facilitate acceptance of ART (68).

According to the recommendations of WHO in developing countries, there is an urgent need for infertility treatment that goes beyond prevention since ART can sometimes be a couple's only option or final resort for getting pregnant, and its demand is particularly high in developing nations. Implementing infertility programs in developing nations requires persuading legislators, health system leaders, policy makers, governmental and non-governmental organizations, religious leaders, community representatives, opinion leaders and also medical industries on the value of infertility care (6, 44, 69). Furthermore, without systematic data on the availability, effectiveness, and safety of reproductive services, it is generally difficult to assess the scope, use and success of ART in a given country and hence it is important to develop and implement appropriate data registry system to follow trend and address gaps (14, 27, 54, 70).

## 5 Limitations

The present systematic review sought to examine the challenges related to ART in developing countries. The main limitation was the difficulty in developing more standardized and singular outcome measures due to the methodological heterogeneity in the approaches used by many researchers to investigate and report on the fundamental questions addressed in our review. Additionally, our review primarily focused on research published in English, which limited our access to studies conducted in other languages from regions where the issue is more prevalent; this may contribute to regional representation bias among the limitations. Notwithstanding the aforementioned limitation this systematic review offers a wealth of evidence to fill the information gap about the difficulties in providing comprehensive care for infertility in a wide range of developing countries. It also outlines significant initiatives that must be implemented to address those challenges on a global and local scale.

## 6 Conclusion

A number of issues that stem from various sources impact the use of ART in developing countries. These include issues related to the high cost of treatment, lack of any form of financial protection or budgeting scheme, accessibility and infrastructure issues, such as scarcity of fertility centers, lack of trained professionals and necessary equipment, and mal distribution of existing ART centers. Other challenges include an absence of policy priority; a lack of clear guidelines, rules, and regulations to govern the sporadically available service; a limited presence of plans and strategies to guide the future direction and current delivery of service; and the existence of negative social, religious, and cultural constructs related use of ART, which further leads to psychosocial distress among users.

Governmental, non-governmental, and international organizations must take a variety of multifaceted and coordinated actions to address those numerous challenges through a short term and long-term plans and strategies. The suggested strategies to deal with financial challenges included government subsidization and incorporation of ART services into the national health insurance scheme in long term, third-party financing, government procurement of ART cycles from private certified facilities, adoption of low-cost ART procedures, coverage of coverage of portion of service costs through cost sharing schemes companies were among the recommendations set out to address the financial challenges based on the finanacil and technical capability of a given developing country.

Distributing fertility centers services fairly by incorporating them in the existing health system, opening additional ART centers, adopting less expensive ART methods, improving referral mechanisms,,, simplifying diagnostic procedures, providing training for healthcare workers, and incorporating infertility treatment into sexual and reproductive health care programs were among prospective recommendations to address infrastructure and accessibility challenges.

Creation and execution of government policies regarding efficient methods for public financing of reproductive care, support for ART regulatory frameworks, inclusion of infertility treatment as a necessary service under universal health coverage, systematic gathering of infertility data by setting up an ART data registry, strengthening the collaboration between the public and private sectors, and moving infertility management higher up the international agenda to help establish adequate funding are among the recommendations forwarded to address policy and political challenges.

Socio-cultural and Psychological challenges can be addressed through the inclusion of client-centered psychosocial interventions as part of ART services and public education to change the societal perception towards ART. To safeguard clients and to maintain sanity in the ART industry, it is also advised that ethical and legal frameworks be developed and the presence of uniform and transparent regulations are ensured. Raising awareness of fertility issues through a public education program and about IVF treatment through targeted patient education among the recommendations to address awareness-related challenges.

## Data Availability

The original contributions presented in the study are included in the article/Supplementary Material, further inquiries can be directed to the corresponding author.
