# Supplementary material for: Systematic review of challenges and prospective recommendations of medically assisted reproductive technology in developing countries
Source: Front Reprod Health. 2025 Nov 27;7:1678033. doi: 10.3389/frph.2025.1678033 (PMC12695750; doi:10.3389/frph.2025.1678033)
Supplement: Supplementary file 2 [file Table2.docx]

Table 2: CASP checklist for risk of bias assessment studies included in a Systematic review of challenges and prospective recommendations of Medically assisted reproductive technology in developing countries. recommendations

|  | Table 2.1: CASP* Checklist for risk of bias assessments for quantitative studies included in a Systematic review of challenges and prospective recommendations of Medically assisted reproductive technology in developing countries. recommendations | | | | | | | | | | | |
| --- | --- | --- | --- | --- | --- | --- | --- | --- | --- | --- | --- | --- |
|  | Reviewers Name: | MDM, DG, LA, VP |  | | | | | | | | | |
| SN | Articles | 1. Was there a clear statement of the aims of the research? | 2.Did the authors use an appropriate method  to answer their question? | 3. Was the research design appropriate to address the aims of the research? | 4. Was the recruitment strategy appropriate to the aims of the research? | 5. Was the data collected in a way that addressed the research issue? | 6. Has the relationship between researcher and participants been adequately considered? | 7. Have ethical issues been taken into consideration? | 8.Was the data analysis sufficiently rigorous? | 9.Is there a clear statement of findings? | 10. How valuable is the research? | Score |
| 1 | Garcia & Bellamy, 2015 | Yes | Yes | can't tell | Yes | Yes | Yes | Yes | Yes | Yes | Yes | 9 |
| 2 | Makuch et al., 2011 | Yes | Yes | Yes | Yes | Yes | can't tell | Yes | Yes | Yes | Yes | 9 |
| 3 | Bennett et al., 2012 | Yes | Yes | Yes | Yes | Yes | Yes | Yes | Yes | Yes | Yes | 10 |
| 4 | Botha et al., 2018 | Yes | Yes | Yes | Yes | Yes | Yes | Yes | Yes | Yes | Yes | 10 |
| 5 | Chiware et al., 2021 | Yes | Yes | Yes | Yes | Yes | Yes | Yes | Yes | Yes | Yes | 10 |
| 6 | Njagi et al., 2023 | Yes | Yes | Yes | Yes | Yes | Yes | Yes | Yes | Yes | Yes | 10 |
| 7 | Fizazi et al., 2022 | Yes | Yes | No | Yes | Yes | Yes | Yes | Yes | Yes | Yes | 9 |
| 8 | Okafor et al., 2017 | Yes | Yes | No | Yes | Yes | Yes | Yes | Yes | Yes | Yes | 9 |
| 9 | Tholeti et al., 2024 | Yes | Yes | Yes | Yes | Yes | Yes | Yes | Yes | No | Yes | 8 |
| 10 | Murage et al., 2011 | Yes | Yes | Yes | Yes | Yes | Yes | Yes | Yes | No | Yes | 9 |
| 11 | Shahin, 2007 | Yes | Yes | Yes | Yes | Yes | Yes | Yes | Yes | Yes | Yes | 10 |
| 12 | Makuch & Bahamondes, 2012) | Yes | Yes | Yes | Yes | Yes | Yes | Yes | Yes | Yes | Yes | 10 |
| 13 | Ma et al., 2023 | Yes | Yes | Yes | Yes | Yes | Yes | Yes | Yes | Yes | Yes | 10 |
| 14 | Bittaye et al., 2023 | Yes | Yes | Yes | Yes | Yes | Yes | Yes | Yes | Yes | Yes | 10 |
| 15 | Majangara Karaga et al., 2023 | Yes | Yes | Yes | Yes | Yes | Yes | Yes | Yes | Yes | Yes | 10 |
| 16 | Dewi et al., 2023 | Yes | Yes | Yes | Yes | Yes | Yes | Yes | No | Yes | Yes | 10 |
| 17 | Chikeme et al., 2022 | Yes | Yes | Yes | Yes | Yes | Yes | Yes | No | No | Yes | 8 |
| 18 | Widge & Cleland, 2009 | Yes | Yes | Yes | Yes | Yes | No | Yes | No | Yes | Yes | 8 |
| 19 | (Afferri et al., 2022 | Yes | Yes | Yes | Yes | Yes | Yes | Yes | Yes | Yes | Yes | 10 |
| 20 | Binarwan Halim E.G., et al, 2020) | Yes | Yes | Yes | Yes | Yes | Yes | Yes | No | Yes | Yes | 9 |
| 21 | Gerrits & Shaw, 2010 | Yes | Yes | Yes | Yes | Yes | Yes | Yes | Yes | Yes | Yes | 10 |

*CASP critical appraisal skill program

**Appraisal Summary**: All quantitative studies included are Positive/Methodologically sound

|  | Table 2.2 CASP Checklist for risk of bias assessment for qualitative studies included in Systematic review of challenges and prospective recommendations of Medically assisted reproductive technology in developing countries. | | | | | | | | | | | |
| --- | --- | --- | --- | --- | --- | --- | --- | --- | --- | --- | --- | --- |
|  | Reviewers Name: | MDM, DG, LA, VP |  | | | | | | | | | |
|  | Articles | 1. Was there a clear statement of the aims of the research? | 2. Is a qualitative methodology appropriate? | 3. Was the research design appropriate to address the aims of the research? | 4. Was the recruitment strategy appropriate to the aims of the research? | 5. Was the data collected in a way that addressed the research issue? | 6. Has the relationship between researcher and participants been adequately considered? | 7. Have ethical issues been taken into consideration? | 8.Was the data analysis sufficiently rigorous? | 9.Is there a clear statement of findings? | 10. How valuable is the research? | Score |
| 1 | Kyei et al., 2020 | Yes | Yes | Yes | Yes | Yes | Yes | Yes | Yes | Yes | Yes | 10 |
| 2 | Souza, 2014 | Yes | Can’t tell | Yes | No | Yes | Yes | Yes | Yes | Yes | Yes | 8 |
| 3 | Hiadzi et al., 2023 | Yes | Yes | Yes | Yes | Yes | No | Yes | Yes | Yes | Yes | 9 |
| 4 | Appiah & Ganle, 2024 | Yes | Yes | Yes | No | Yes | Yes | Yes | Yes | Yes | Yes | 9 |
| 5 | Bezad et al., 2022 | Yes | Yes | Yes | Yes | Yes | Yes | Yes | Yes | Yes | Yes | 10 |
| 6 | Afferri et al., 2024 | Yes | Yes | Yes | Yes | Yes | No | Yes | Yes | Yes | Yes | 9 |
| 7 | Purvis, 2015 | Yes | Yes | can't tell | can't tell | Yes | Yes | Yes | Yes | Yes | Yes | 8 |
| 8 | Asante-Afari et al., 2022 | Yes | Yes | Yes | Yes | No | Yes | Yes | Yes | Yes | Yes | 9 |
| 9 | Inhorn, 2003 | Yes | Yes | can't tell | Yes | Yes | Yes | Yes | Yes | Yes | Yes | 9 |
| 10 | Akande et al., 2019 | Yes | Yes | No | Yes | Yes | Yes | Yes | Yes | Yes | Yes | 8 |
| 11 | Barnes et al., 2024 | Yes | Yes | Yes | Yes | Yes | Yes | Yes | Yes | Yes | Yes | 10 |
| 12 | Anaman-Torgbor et al., 2021 | Yes | Yes | Yes | Yes | Yes | Yes | Yes | Yes | Yes | Yes | 10 |
| 13 | Ombelet et al., 2008 | Yes | Yes | Yes | Yes | Yes | Yes | Yes | Yes | Yes | Yes | 10 |
| 14 | Okantey, 2021 | Yes | Yes | Yes | Yes | Yes | Yes | Yes | Yes | Yes | Yes | 10 |
| 15 | Ezeome et al., 2023 | Yes | Yes | Yes | Yes | Yes | Yes | Yes | Yes | Yes | Yes | 10 |
| 16 | Whittaker et al., 2024 | Yes | Yes | Yes | Yes | Yes | Yes | Yes | Yes | Yes | Yes | 10 |
| 17 | Z Ahmed Murad et al., 2014 | Yes | No | Yes | Yes | Yes | No | Yes | Yes | No | Yes | 10 |
| 18 | Oti-Boadi et al. 2024 | Yes | Yes | Yes | No | can't tell | Yes | Yes | Yes | Yes | Yes | 8 |
| 19 | Ranjbar et al., 2015 | Yes | Yes | Yes | Yes | Yes | Yes | Yes | Yes | No | Yes | 9 |
| 20 | Gerrits & Shaw, 2010 | Yes | Yes | can't tell | Yes | Yes | Yes | Yes | No | No | Yes | 7 |
| 21 | Dyer et al., 2017 | Yes | Yes | Yes | Yes | Yes | Yes | Yes | Yes | Yes | Yes | 10 |
| 22 | Njogu et al., 2022 | Yes | Yes | Yes | Yes | Yes | Yes | Yes | Yes | Yes | Yes | 10 |

*CASP critical appraisal skill program

**Appraisal Summary**: All qualitative studies included are Positive/Methodologically sound
