# Supplementary material for: Systematic review of challenges and prospective recommendations of medically assisted reproductive technology in developing countries
Source: Front Reprod Health. 2025 Nov 27;7:1678033. doi: 10.3389/frph.2025.1678033 (PMC12695750; doi:10.3389/frph.2025.1678033)
Supplement: Supplementary file 3 [file Table3.docx]

Table 3: Summary of articles included in the systematic review of challenges and prospective recommendations of Medically assisted reproductive technology in developing countries

| S. N | Studies | Region | Country /Region | Population | Study design | Major challenges associated with assisted reproductive technology | | | | | | |
| --- | --- | --- | --- | --- | --- | --- | --- | --- | --- | --- | --- | --- |
|  |  |  |  |  |  | Affordability  /Cost | Accessibility/  Infrastructural | Socio-cultural | Ethico  -legal | Religious | Political | Awareness |
| 1 | Kyei et al., 2020 | Africa | Ghana | Infertility Clients | Qualitative | **√** | **√** | **√** |  |  |  |  |
| 2 | Dyer et al., 2020 |  | Africa | Art Centers | Data registry |  | **√** |  |  |  | **√** |  |
| 3 | Appiah & Ganle, 2024 |  | Ghana | Art Professionals | Qualitative | **√** | **√** | **√** | **√** |  |  |  |
| 4 | Hiadzi et al., 2023 |  | Ghana | Infertility Clients, Providers | Qualitative | **√** | **√** |  |  |  |  |  |
| 5 | Bezad et al., 2022 |  | Morocco | Policymakers, Health Actors | Qualitative |  |  |  |  |  | **√** |  |
| 6 | Afferri et al., 2024 |  | Gambia | Policymakers, Practitioners | Qualitative Survey | **√** | **√** |  |  |  | **√** |  |
| 7 | Botha et al., 2018 |  | SSA | Clients, Registries | Systematic review | **√** |  |  |  |  | **√** |  |
| 8 | Fizazi et al., 2022 |  | Algeria | Infertility Clients | Quantitative survey | **√** |  |  |  |  |  |  |
| 9 | Asante-Afari et al., 2022 |  | Ghana | Infertility Clints | Qualitative Survey | **√** |  |  |  | **√** |  |  |
| 10 | Inhorn, 2003 |  | Egypt | Provides, Clients | Review |  |  | **√** |  |  |  |  |
| 11 | Okafor et al., 2017 |  | Nigeria | Infertility Clients | Cross-sectional | **√** |  |  |  |  | **√** |  |
| 12 | Akande et al., 2019 |  | Nigeria | Infertility Clients | Cross-sectional | **√** |  |  |  |  |  | **√** |
| 13 | Barnes et al., 2024 |  | Ghana | Professionals, Managers, | Qualitative |  | **√** |  | **√** |  | **√** |  |
| 14 | Anaman-Torgbor et al., 2021 |  | Ghana | Infertility Clients | Qualitative | **√** |  | **√** |  |  |  |  |
| 15 | Murage et al., 2011 |  | Kenya | Obstetricians, Gynecologists | Cross-sectional | **√** | **√** |  |  |  |  |  |
| 16 | Shahin et al, 2007 |  | Egypt | Infertility Clients | Quantitative survey | **√** |  |  |  |  |  |  |
| 17 | Okantey et al, 2021 |  | Ghana | Infertility Clients, Professionals | Qualitative | **√** |  | **√** |  |  |  |  |
| 18 | Ezeome et al., 2023 |  | Nigeria | ART Clients | Qualitative | **√** |  | **√** |  |  |  | **√** |
| 19 | Whittaker et al., 2024 |  | South Africa, Zimbabwe, | ART Specialists, Gynecologists | Qualitative | **√** | **√** |  |  |  | **√** |  |
| 20 | Bittaye et al., 2023 |  | Gambia | Professionals, Students | Quantitative survey |  | **√** |  | **√** | **√** | **√** |  |
| 21 | Majangara Karaga et al., 2023 |  | SSA | Clinicians, Authorities | Mixed Methos | **√** |  |  |  |  | **√** |  |
| 22 | Oti-Boadi et al. 2024 |  | Ghana | Infertility Clients | Qualitative Survey | **√** |  | **√** |  |  |  | **√** |
| 23 | Chikeme et al., 2022 |  | Nigeria | Infertility Clients | Cross-sectional Survey | **√** | **√** |  |  |  | **√** | **√** |
| 24 | Afferri et al., 2022 |  | Gambia | Health Facilities | Cross-sectional study | **√** | **√** |  |  |  | **√** |  |
| 25 | Gerrits & Shaw, 2010 |  | SSA | Professionals, Clients | Systematic review |  | **√** | **√** |  |  |  |  |
| 26 | Dyer et al., 2017 |  | South Africa | ART Clients | Observational follow-up study | **√** |  |  |  |  | **√** |  |
| 27 | Njogu et al., 2022 |  | Kenya | ART Clients | Qualitative | **√** |  | **√** |  |  |  |  |
| 28 | Purvis, 2015 | Asia | Indonesia | Provides, Clients | Review | **√** |  |  |  |  |  |  |
| 29 | Tholeti et al., 2024 |  | India | Provides, Clients | Review | **√** |  |  |  |  |  |  |
| 30 | Ma et al., 2023 |  | China | Registries | Cross-sectional | **√** | **√** |  |  |  |  |  |
| 31 | Z Ahmed Murad et al., 2014) |  | Malaysia | Infertility Clients, Professionals | Qualitative |  |  |  |  | **√** |  |  |
| 32 | Widge & Cleland, 2009 |  | India | Gynecologists | Cross-sectional survey |  | **√** |  |  |  | **√** |  |
| 33 | Binarwan H.et al, 2020 |  | Indonesia | ART Clients | Quantitative survey | **√** | **√** |  |  |  |  |  |
| 34 | Bennett et al., 2012 |  | Indonesia | Infertility clients | Quantitative Survey |  | **√** |  |  |  |  |  |
| 35 | Ranjbar et al., 2015 | Gulf | Iran | Infertility Clients | Qualitative |  |  | **√** |  |  |  |  |
| 36 | Makuch & Bahamondes, 2012 | Latin America | Brazil | Health Authorities | Mixed Methos | **√** | **√** |  |  |  | **√** |  |
| 37 | Garcia & Bellamy, 2015 |  | Brazil | Art Centers | Qualitative | **√** | **√** |  | **√** | **√** |  |  |
| 38 | Souza, 2014 |  | Brazil | Art Centers | Qualitative | **√** |  |  |  |  |  |  |
| 39 | Makuch et al., 2011 |  | Brazil | Practitioners, infertility clients | Qualitative | **√** | **√** |  |  |  |  |  |
| 40 | Chiware et al., 2021 | Global | LMIC | Clients, Registers | Systematic review | **√** | **√** |  |  |  |  |  |
| 41 | Njagi et al., 2023 |  | LMIC | Provides, Clients | Systematic review | **√** |  |  |  |  | **√** |  |
| 42 | Ombelet et al., 2008 |  | Developing countries | Professionals, Clients | Systematic review |  | **√** |  |  |  | **√** |  |
| 43 | Dewi et al., 2023 |  | developing countries | Provides, Clients | Scoping review |  | **√** | **√** |  | **√** |  | **√** |

(√: challenges mentioned in the study)
