# Supplementary material for: Systematic review of challenges and prospective recommendations of medically assisted reproductive technology in developing countries
Source: Front Reprod Health. 2025 Nov 27;7:1678033. doi: 10.3389/frph.2025.1678033 (PMC12695750; doi:10.3389/frph.2025.1678033)
Supplement: Supplementary file 4 [file Table4.docx]

| Table 4: Summary of challenges and prpspective recommendations from articles included in the systematic review of challenges and prospective recommendations of Medically assisted reproductive technology in developing countries | | | |
| --- | --- | --- | --- |
| **S. N** | **Reference** | **Challenges** | **Prospective recommendations** |
| 1 | Kyei et al., 2020 | High cost of ART treatment, anxiety, disruptions of daily life, and long commutes to treatment centers were among the major challenges identified. | The authors recommended insurance companies should cover a portion of the cost and that counseling services be integrated into ART centers. |
| 2 | Dyer et al., 2020 | Based on a study that examined trends in ART usage in Africa between 2013 and 2017. The rate of ART use was uniformly low in all countries. Although the statistics are not yet representative, the strongest evidence suggests that access to ART is restricted. |  |
| 3 | Souza, 2014 | Treatment costs were significant challenges that continued to be beyond the means of treatment for the vast majority of Brazilians. | Government purchasing ART cycles from private certified facilities and insurance coverage to provide ART procedures at low cost to low-income populations were the recommendations. |
| 4 | Garcia & Bellamy, 2015 | According to a study conducted in the Brazilian health system to look at access to reproductive technologies, the majority of assisted reproductive technologies are provided by private clinics at high prices, and there is no particular legislation governing ART services in Brazil. |  |
| 5 | Hiadzi et al., 2023 | In urban Ghana, clients need to ensure that they have enough money to pay for ART and also experience moral dilemmas over the use of reproductive services. | Third-party funding, patient education, society participation, and continuous ART monitoring were among the recommendations. |
| 6 | Appiah & Ganle, 2024 | The practice of in vitro fertilization (IVF) in Ghana raises a number of ethical issues, including decisions regarding the management of surplus embryos and unequal access to care due to high costs and limited availability. | The creation of uniform and transparent regulations to regulate the practice of IVF was the forwarded solution. |
| 7 | Bezad et al., 2022 | Among other things, there were insufficient political entrepreneurs to guarantee capable leadership; political windows were frequently overlooked; and community members could not agree on a clear public presentation of the assisted reproduction. | Translation of ART regulations into measurable activities, high-quality fertility care, and equitable fertility treatment coverage to address the needs of infertile couples were among the recommendations. |
| 8 | Makuch et al., 2011 | According to the perspectives of Brazilian health professionals with respect to access to ART procedures, high treatment costs and long waiting times were major challenges | Development of a strategy to make ART more affordable and available to a significant portion of the population was deemed essential. |
| 9 | Afferri et al., 2024 | Absence of financial protection mechanisms for infertile couples, Lack of routinely collected infertility data, gaps in fertility care training among health practitioners, and limited cooperation between the public and private sectors in the provision of fertility care were major barriers. | Systematic gathering of infertility data, raising awareness of fertility issues, and offering specialized training and strengthening the collaboration between the public and private sectors were prospective solutions |
| 10 | Bennett et al., 2012 | High physician change rates and low confidence in infertility therapy, high treatment costs, a lack of a reliable referral system, and an insufficient number and location of ART clinics were among the challenges. | Four recommendations were set to address the challenges: - More patient education, funding for infertility clinics, advancements in the cost-effectiveness of infertility treatment, and improvement in referral mechanisms |
| 11 | Botha et al., 2018 | The availability and utilization of ART are extremely low in sub-Saharan Africa, according to evidence regarding its efficacy and safety. There is currently only one national registry and no regional registry, and the primary obstacle to service access is the high cost of ART | One potential way to build evidence-based practice is to set up a regional ART data registry to report and track the availability, efficacy, and safety of ART. |
| 12 | Chiware et al., 2021 | The purpose of this review was to examine the current state of affordable infertility treatment options that are being developed. The results demonstrated that no LMIC research had documented the use of Low-cost ART that is efficient, available, and within the means of the majority of individuals. | The authors recommended WHO to create and disseminate the necessary guidelines with that regard |
| 13 | Njagi et al., 2023 | Findings from the review demonstrated that clients' direct medical expenses for infertility treatment are far greater than the population's yearly average income and GDP per capita, making them unaffordable and putting those in need at danger of catastrophic spending. | Creation and execution of government policies regarding efficient methods for public financing of reproductive care, support for ART regulatory frameworks and inclusion of infertility treatment as a necessary service under universal health coverage were the solutions forwarded. |
| 14 | Fizazi et al., 2022 | Exorbitant costs for ART and the National Social Security Fund's failure to refund this act were the main issues found in ART sector of Algeria. | Inclusion of ART the National Social Security Fund repaying some ART procedures or providing the ART units with the tools required for IVF and ICSI procedures were recommendations set forth. |
| 15 | Purvis, 2015 | Religious and economic factors have a bigger influence on the clinical application of ART than official laws. Furthermore, ART policies created by fertility clinics are more stringent than those mandated by law. High cost of infertility treatment and regional variations in healthcare delivery were another challenge for Indonesians. | Indonesia can do a lot to increase access to ART generally by distributing services fairly, creating less expensive ART methods, and encouraging STI prevention and detection. |
| 16 | Asante-Afari et al., 2022 | The way society perceives children born after assisted reproductive technology has caused anxiety in women who have given birth after using it. | Public education needed to change the societal perception towards women who utilize assisted reproductive technologies. |
| 17 | Inhorn, 2003 | In as Egypt major challenge of ART was in the areas of cost and feasibility the reason was lack of government willingness to commit state resources on the provision of NRTs. | The necessity for primary prevention of infertility, specifically early and efficient treatment of reproductive tract infections, was the most prominent recommendation. |
| 18 | Okafor et al., 2017 | One of the main Challenges influencing how participants view IVF is a lack of knowledge about it. The high expense of IVF therapy was another significant obstacle. | Government subsidization of cost insurance coverage and raising awareness about IVF treatment were among the prospects. |
| 19 | Akande et al., 2019 | A low level of willingness to use ART and a negative attitude regarding its use and expensiveness were shared challenges by infertile people in Nigeria. | A public education program, opening additional ART centers, and lowering the cost of the services were among the recommendations. |
| 20 | Tholeti et al., 2024 | Dominance of the private sector, high cost of treatment cycles, and lack of insurance coverage are among the major challenges in the Indian healthcare system that limit couples' ability to undergo fertility treatment. | Policy reforms, increased public awareness, and the development of affordable treatment options are required for broader access. |
| 21 | Barnes et al., 2024 | Absence of ethical and legal frameworks for ART practice, ethical challenges existing in the areas of informed consent, clients’ privacy and clinical data protection, and gamete donation were among challenges in ART service provision in Ghana. | To safeguard clients and to maintain sanity in the ART industry, it is advised that ethical and legal regulatory frameworks be developed. |
| 22 | Anaman-Torgbor et al., 2021 | Women seeking ART services in Ghana experience anxiety, stress, frustration and are burdened with the challenge of funding the treatment. | Inclusion of client-centered psychosocial interventions as part of ART services and complementary health insurance coverage recommended to address multi- dimensional challenges |
| 23 | Murage A. et al, 2011 | In Kenya, ART service provision was severely limited to only three units, despite the reported high rate of tubal disease. The high cost of treatment and limited local services were almost universally cited as the main challenges. | To address the aforementioned issues, it was recommended that simpler, less expensive, and more accessible ART methods be taken into consideration. |
| 24 | Shahin, 2007 | High cost of the ART service was cited as a significant obstacle by infertility clients in Egypt. | Moving infertility management higher up the international agenda to help establish adequate funding policies is recommended. |
| 25 | Makuch & Bahamondes, 2012 | In Brazil, over 63% of the authorities stated that infertility treatments were not readily available. Lack of financial resources and political decisions to implement them were among the challenges, in addition to lengthy wait times, intricate scheduling procedures, and a lack of initiative to introduce less expensive ARTs, which were among the obstacles. |  |
| 26 | Ombelet et al., 2008 | Lack of registration system and priority for ART among most international NGOs were the identified gaps. | Simplification of diagnostic and ART procedures, providing training for healthcare workers, and incorporating infertility treatment into sexual and reproductive health care programs were prospective recommendations. |
| 27 | Ma et al., 2023 | A low number of ART clinics and the absence of subsidized infertility treatments through the national health insurance scheme were among the challenges in China. | Besides increasing the number of ART clinics, devising cost-effective treatment to improve the accessibility and availability was among the recommendations forwarded. |
| 28 | Okantey, 2021 | Further to the high cost of treatment, individuals who resort to ART to have children are faced with a myriad of psychosocial problems, stigma, and discrimination, as those discriminations do not exclude the children that are born from ART in Ghana | It was advised that health professionals provide couples therapy to lessen the psychological impact, and public education and sensitization campaigns are also required to raise knowledge of ART. |
| 29 | Ezeome et al., 2023 | A study from Nigeria showed that most of the customers came to their ART providers at an advanced age due to knowledge gaps and the expensive cost of ART services. Feelings of stigmatization and embarrassment both during and after the procedure are also another gap. | Government subsidization and incorporation of ART services into the National Health Insurance Scheme were recommended to address the cost challenge |
| 30 | Whittaker et al., 2024 | Lack of ART availability was caused by a number of factors, including a lack of public money, the high cost of treatment, a lack of priority, a lack of policy awareness, a lack of ART clinics and highly trained expert staff, and overservicing in the primarily privatized sector in several African nations. | Authors of the study recommended that the government need to subsidize assisted reproduction in the public sector. |
| 31 | Z et al., 2014 | A Malaysian study to ascertain whether ARTs are Shari’aa-compliant found that while participants were reasonably aware of some of the fatwas currently in effect regarding ART, there were still certain questions that needed to be addressed. | The establishment of ART services that are demonstrably Shari'aa compliant was recommended. |
| 32 | Bittaye et al., 2023 | Lack of infertility services in the rural areas of the country, competing importance of other health priorities, religious and cultural barriers, and lack of formal infertility training among most medical staff were challenges to introducing ART in Gambia. | Expansion of infertility services and the supply of specialized infertility training for medical professionals were among the recommendations. |
| 33 | Majangara Karaga et al., 2023 | The presence of copayments for ART services, lack of policy and legislation, high costs, and bureaucratic obstacles were among the leading challenges in the delivery of ART in the public sector of Africa. | Development of appropriate’ policy, funding strategy and good health service infrastructure were suggested as main enablers to improve ART service. |
| 34 | Oti-Boadi et al. | An assessment of the cultural and religious beliefs about ART in Greater Accra, Ghana, showed that despite their high awareness regarding ART, clients faced some challenges while attempting to use the service, such as difficulty getting a surrogate mother, high cost of ART services, stigmatization, and fear of contracting HIV. |  |
| 35 | Dewi et al., 2023 | A review of IVF access barriers found that the main obstacles to participating in IVF programs were limited access, low government policy priority, a lack of ART centers, a lack of qualified infertility-trained staff, a lack of government support, and sociocultural factors. | The way out to such challenges is to start an IVF program that was acceptable, economical and successful. |
| 36 | Ranjbar et al., 2015 | Four main themes revolving around ART in Iran were uncovered, including a struggle to achieve pregnancy, fear and uncertainty, escape from stigma, and the pursuit to achieve husband satisfaction. | Establishment and integration of counseling services for clients enrolling in ART and involvement of partners in counseling sessions were recommended. |
| 37 | Chikeme et al., 2022 | Results from a Nigerian study of women who visited reproductive clinics revealed that consumers' awareness of ARTs was inadequate and they had poor levels of service utilization and treatment efficacy perception. One of the biggest obstacles was identified as the procedure's high cost. | The research paper suggests cost-cutting initiatives and public awareness campaigns. |
| 38 | Widge & Cleland, 2009 | Geographic inequity, commercialization of service, variable quality, clients lack of knowledge, high cost, unnecessary repetition of investigation, low success rates, lack of clear selection criteria, lack of specialized training, deficiency in record-keeping, lack of counseling service, and lack of transparency were among the challenges in the Indian ART survey. | Appropriate monitoring and regulation shall be in place to address the challenges in place. |
| 39 | Afferri et al., 2022 | Gambia's infertility services availability trend is comparable to that of other sub-Saharan African nations where the private sector dominates service and inaccessibility to care is worsened by the high cost and geographic limitations. | Organized monitoring of infertility data and funding of reproductive service packages were among the recommendations |
| 40 | Binarwan Halim | Geographical and sociocultural barriers were more common in Indonesian rural areas than in urban areas when comparing ART service hurdles for consumers in rural and urban locations. | Potential remedies included lowering medical expenses, enhancing the referral system, and utilizing telemedicine. |
| 41 | Gerrits & Shaw, 2010 | No report has indicated the availability of quality fertility service care that is offered in a standardized way in the public health system of sub-Saharan Africa. In addition, ARTs are mostly available at private providers at exorbitant prices. | It is recommended that health personnel have proper training and that counseling procedures be improved at all levels of the healthcare system. |
| 42 | Dyer et al., 2017 | The financial recovery rate after ART was less than half, and the ability to pay is not always implied by a patient's willingness to pay for ART, according to a study on the financial recovery of households. Absence of comprehensive third-party funding for ART can lead to both short-term and long-term financial difficulties. | Promotion of financial risk protection and comprehensive third-party funding for ART were the solutions forwarded. |
| 43 | Njogu et al., 2022 | According to a study that investigated how infertile women handled the demands of infertility therapy, it was discovered that therapy was financially limiting, physically difficult, and emotionally upsetting. Their husbands, families, and friends suffered as a result of fertility treatment. | Integrating counseling services with ART and educating society about infertility therapy were among prospective solutions. |

(ART: Assisted reproductive technology, GDP: Gross domestic product, ICSI: Intra cytoplasmic sperm injection, IVF: Invitro Fertilization, LMIC: Low- and middle-income country, MART: Medically Assisted reproductive technology, NGOs: Non-governmental organizations, NRTs: New reproductive technologies, STI: Sexually transmitted infection, SSA: Sub-Saharan Africa, WHO: World health organization)
