# Supplementary material for: Systematic review of challenges and prospective recommendations of medically assisted reproductive technology in developing countries
Source: Front Reprod Health. 2025 Nov 27;7:1678033. doi: 10.3389/frph.2025.1678033 (PMC12695750; doi:10.3389/frph.2025.1678033)
Supplement: Supplementary file 5 [file Datasheet1.zip › Figure 2_Frequency of major challenges.docx]

Figure 2. Frequency of major challenges associated with assisted reproductive technologies in developing countries
