# Supplementary material for: Systematic review of challenges and prospective recommendations of medically assisted reproductive technology in developing countries
Source: Front Reprod Health. 2025 Nov 27;7:1678033. doi: 10.3389/frph.2025.1678033 (PMC12695750; doi:10.3389/frph.2025.1678033)
Supplement: Supplementary file 5 [file Datasheet1.zip › Figure 3_Summary of challenges.docx]

**Socio-cultural & Psycho-Social**

- Stigmatization & discrimination
- Negative view towards ART born children
- Absence of spouse support/satisfaction
- Negative attitude towards the use of ART services
- Negative moral judgment
- Higher level of Depression and anxiety
- Emotional exhaustion
- Experience of fear and uncertainty
- escape from stigma
- Difficulty of disclosing information
- Negative relation with husbands, family, and friends
- perceived treatment failure

**Financial**

- Very high cost of ART
- Absence of financial protection mechanisms
- lack of specific budget for infertility
- Risk of catastrophic expenditure
- Insufficient funds
- Poor financial recovery

Policy /Political

- Poor policy awareness and prioritization of fertility problems
- Bureaucratic obstacles
- Lack of Monitoring and regulation of service
- Absences of public private partnership
- Lack of policy and guidelines
- Absence of national. Regional plan
- Absence strong leadership
- Absence of national registry
- Lack of willingness to commit state resources

**Accessibility/Infrastructural**

- Geographically restricted
- Long distance traveling
- Long waiting time
- Poor referral system
- Disturbances in daily routine and work
- shortage of infertility centers
- Privatization & commercialization
- low quality service
- Unnecessarily repetition of investigations
- Absence’s counselling and lack transparency
- Lack rigorous specialized training
- Deficient in record-keeping
- Shortage of trained human resources

**Ethico-legal**

- Moral dilemmas regarding embryo
- Inequitable access
- Poor clients’ privacy and clinical data protection
- Lack informed consent
- Gamete donation issues
- Absence of Ethical guidelines

**Religious**

- Myths and beliefs against ART & infertility
- Restriction of treatment modalities
- Incompatibility with existing religious laws
- Discrimination against users

Figure 3: Summary of challenges associated with Medically Assisted Reproductive Technology in developing countries: (ART: assisted reproductive technology)
